# BRICS soft power promotion: Dataset for media preference and use pattern among the Russian audience who follow the development of BRICS

**DOI:** 10.1016/j.dib.2017.12.004

**Published:** 2017-12-20

**Authors:** N. Zavyalova, E.M. Akhmetshin

**Affiliations:** a Ural Federal University, 620002, 19 Mira Street, Ekaterinburg, Russia; b Kazan (Volga region) Federal University, Elabuga Institute of KFU, 423604, 89, Kazanskaya Street, Elabuga, Russia

**Keywords:** Communication, Format, Strategy, Soft power, Rising power

## Abstract

The focal point of the paper is an attempt to identify the most effective way to offer the information about BRICS-related subjects. BRICS organization has already become the topical issue of our research. This paper highlights the research of the best way to deliver BRICS messages. Communication plays a pivotal role in our present-day life. It is essential for a wide range of technologies. Therefore, our study has several objectives, i.e., to describe the difference between the Internet-oriented communication and the communication supported by television and press. These data may be useful for a more effective coverage of BRICS-related subjects. Another important objective is the analysis of implied information given on official BRICS-related websites. The data collection procedure is as follows. The central concern of the paper is the best media format which we tried to calculate using the data from official BRICS summit websites in Russia and China. These data are compared with the results of our own survey which we conducted with the help of a specially designed questionnaire. The experiment does not include any ethical issues but is purely descriptive in its nature and contains the information about data processing. The target audience of the research are professionals engaged in the field of BRICS news coverage, BRICS analytics and BRICS expertise.

**Specifications Table**

**Data set 1**

**Table:** 

Subject area	Cognitive Psychology
More specific subject area	Linguistics and communication
Type of data	8 graphs
How data was acquired	Official website of Russia's presidency in BRICS
	Official website of China's presidency in BRICS
Data format	**Networks generated by Cytoscape version 3.5.1. given in.jpeg images**
**Experimental factors**	**key messages**
**Experimental features**	The data helps see the communicative aspects of BRICS-strategies and helps communicate themes of BRICS more efficiently, according to a certain brain map.
	The information is relevant for experts working in the sphere of political journalism, blogging and mass media.
Data source location	http://en.brics2015.ru/multimedia/
	https://www.brics2017.org/English/China2017/Logo/
	https://www.brics2017.org/English/China2017/Theme/
Data accessibility	The data are available as open data license

**Data set 2**

**Table:** 

Subject area	Decision making and information
More specific subject area	Communication and politics
Type of data	Numeric variables
How data was acquired	Surveys through face to face interviews were conducted using a structured questionnaire
Data format	**4 tables**
**Experimental factors**	**opinions**
**Experimental features**	The data helps understand the correlation between the media preference and use pattern
Data source location	Urals, Russia
Data accessibility	The data are included in this article

**Value of the data**

This data provides insights for a deep understanding of the following research areas:

• The communicative shifts in BRICS promotion.
• The description of Russian communicative media users in terms of their media preferences and in terms of time and the duration of media use.
• The preferences of media users and how they are changing.
• Why effective communication is the next frontier for BRICS think tanks?
• The evaluation of hurdles which may prevent BRICS-oriented audiences from getting the message of key players.
• The ways to overcome these hurdles with the help of different media communication formats.
• The potential of long-run communicative strategies.

Our central hypothesis is that each logo of the summit should be given in a format which directly correlates with the best-preferred format of BRICS-audiences.

## 1 Data

There is a growing body of literature which recognizes the importance of successful communication. BRICS organization has already become the topical issue of our research (Zavyalova, 2017) [4], and (Zavyalova, 2017) [5]. We collected the data which describes BRICS-related discussion.

The first data set is composed of infographic images from Russian presidency in BRICS in 2015 official website [1] and Chinese presidency in BRICS in 2017 official website [2], [3] generated in the form of networks by Cytoscape version 3.5.1. The striking difference between these two sets of infographic images is the communicative shift which is suggestive of differences in overall policies of president countries.

The first series of infographic images stresses the value of each member-state, the uniqueness and individual stake within BRICS framework. The profile of each country is supported by descriptive statistics highlighting economic and cultural advantages of each country [7], [8]. Our understanding is that this series of images works best for the Internet-oriented users. However, if we consider the countries where the Internet is not still well-developed, this format is not easy. It does not offer a single clear-cut image.

The logo of the whole year of Russian presidency in BRICS in 2015 resembles a colorful mosaic which puts forward the idea of unique contributions of each member-state, yet highlights the ensemble effort. It is a powerful solution for the Internet-audience because each letter is interactive and connected with other pages providing additional information about each country.

However, Chinese BRICS summit in Xiamen in 2017 logo stresses the idea of mutual effort. Here is the official description of the logo, “The main component of the 2017 BRICS Summit Logo resembles both full sails and a rotating earth, painted in 5 colors representing the 5 BRICS countries. The symbolism is two-fold: BRICS countries breaking the waves in the same boat towards a brighter future; and BRICS playing an important role in global political and economic affairs. The Logo brings out the theme of the Summit ”BRICS: Stronger Partnership for a Brighter Future”, and brims with the distinct marine culture of the host city Xiamen. At the bottom are the English words BRICS 2017 CHINA and a red seal reading China, highlighting China's BRICS Chairmanship for 2017. The seal is engraved in Big Seal Script or Dazhuan, an ancient Chinese calligraphic style, giving the logo a specific touch of the traditional Chinese culture’’ [2].

The comparison of the two image sets result in the idea that year after year BRICS is gaining momentum in communicating new messages: from individual efforts to a more cooperative united endeavor, from the message of BRICS mixed nature to the message of unity. Our next question is whether BRICS communicative strategy is successful and whether it reaches the audience (Pic. 1, Pic. 2, Pic. 3, Pic. 4, Pic. 5, Pic. 6, Pic. 7, Pic. 8).

**Pic. 1:**
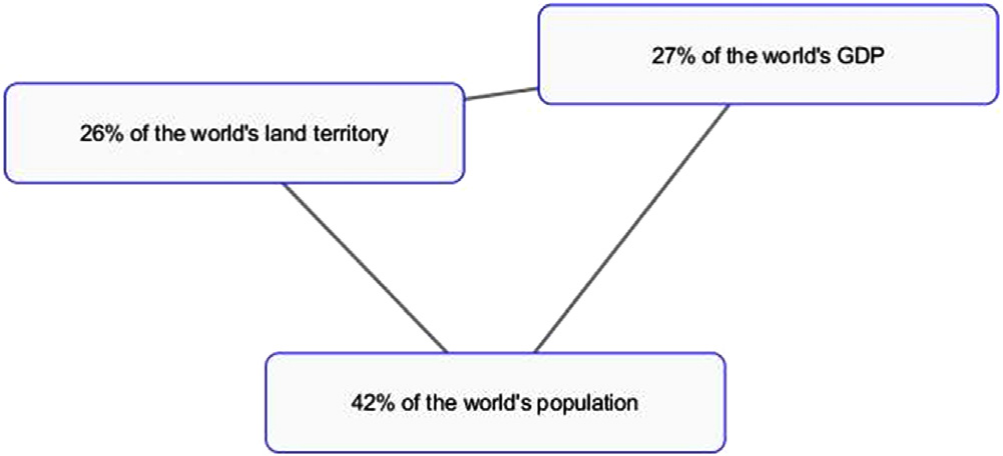
Infographics “BRICS in Numbers” [1].

**Pic. 2:**
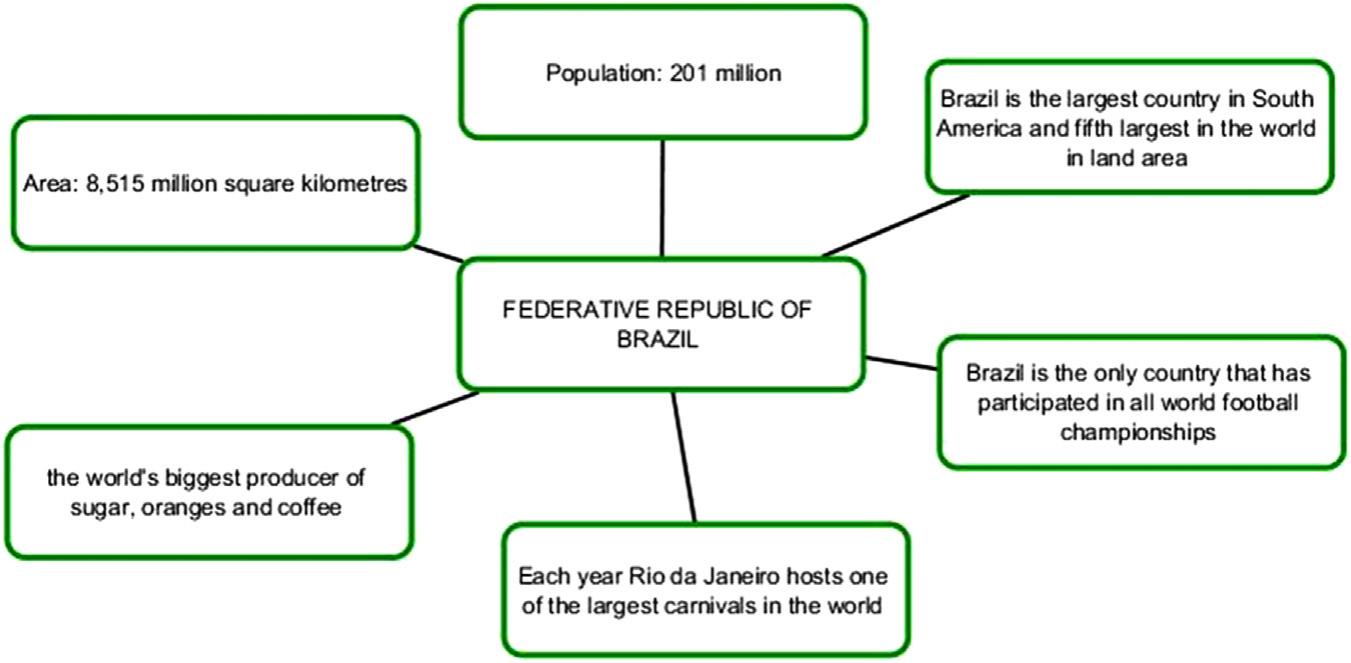
Infographics “Federative Republic of Brazil” [1].

**Pic. 3:**
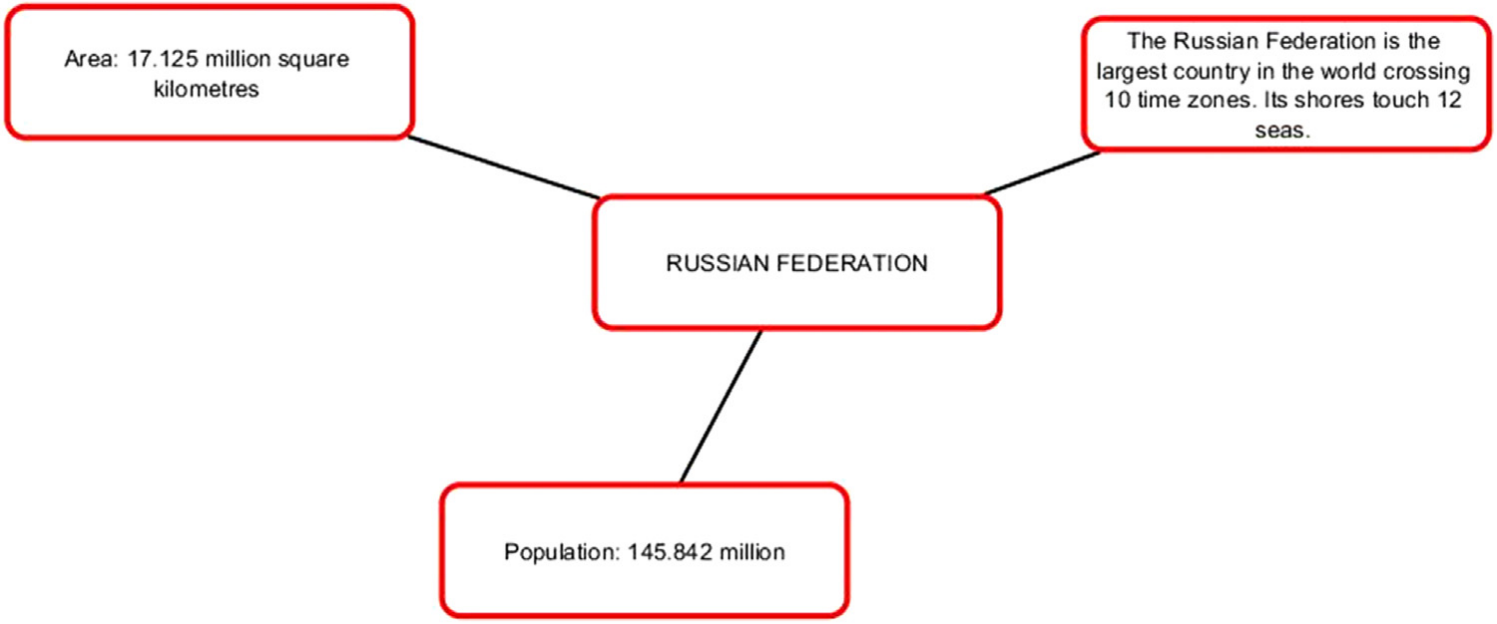
Infographics “Russian Federation” [1].

**Pic. 4:**
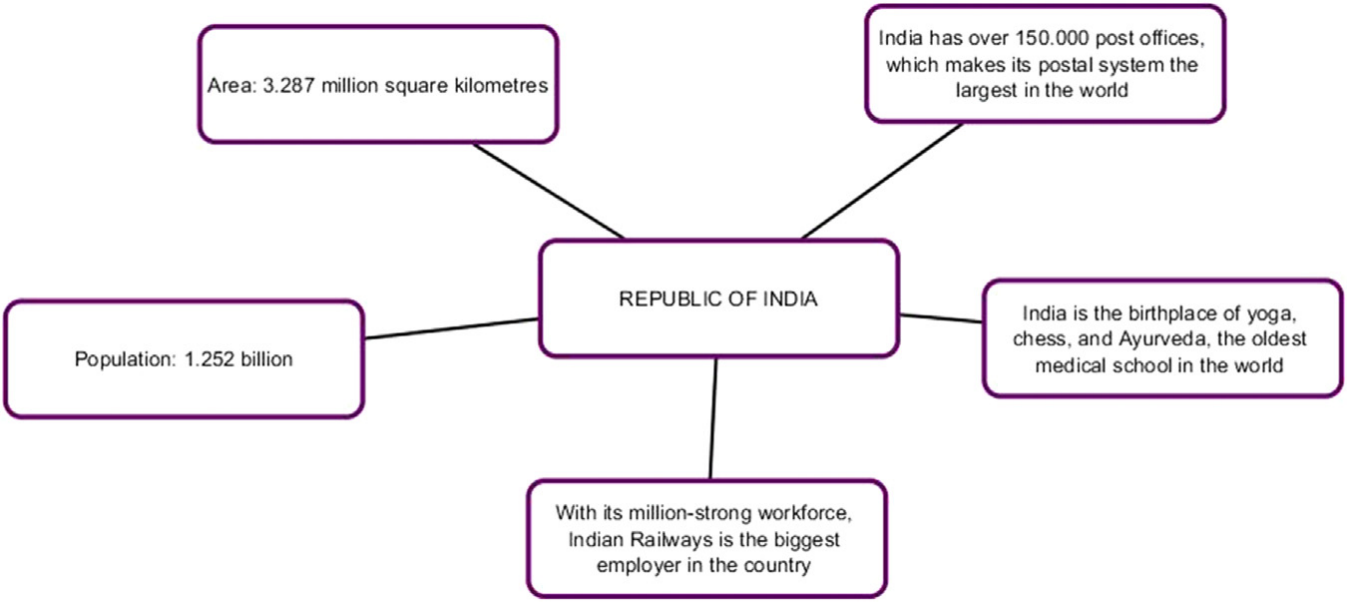
Infographics “Republic of India” [1].

**Pic. 5:**
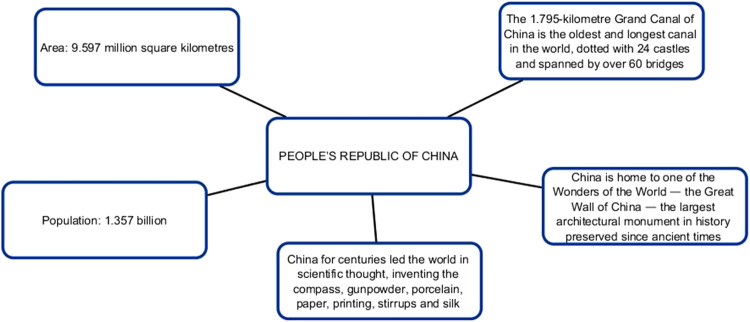
Infographics “People's Republic of China” [1].

**Pic. 6:**
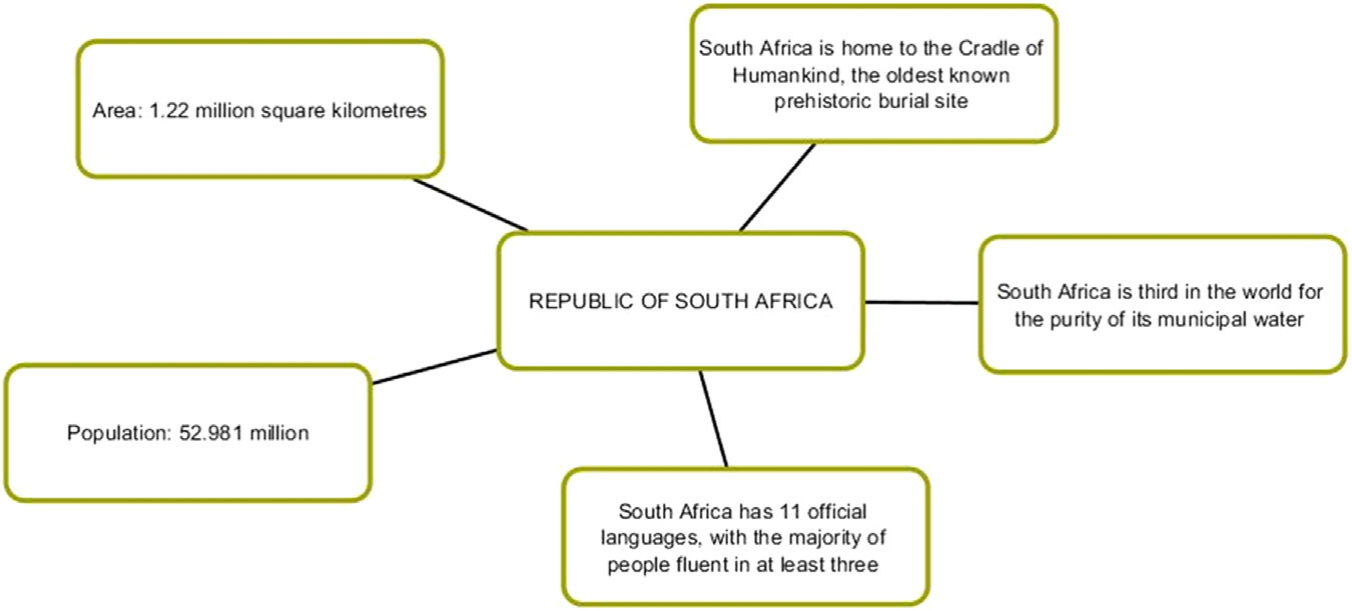
Infographics “Republic of South Africa” [1].

**Pic. 7:**
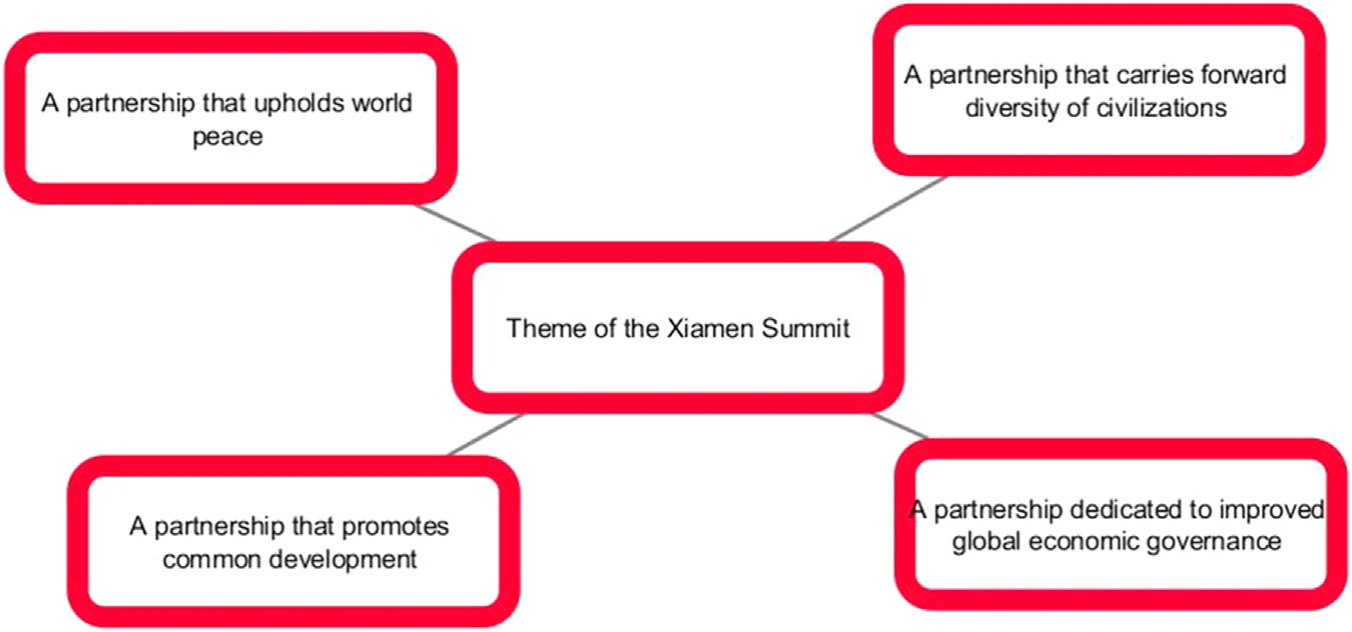
The theme of Xiamen summit in 2017 [3].

**Pic. 8:**
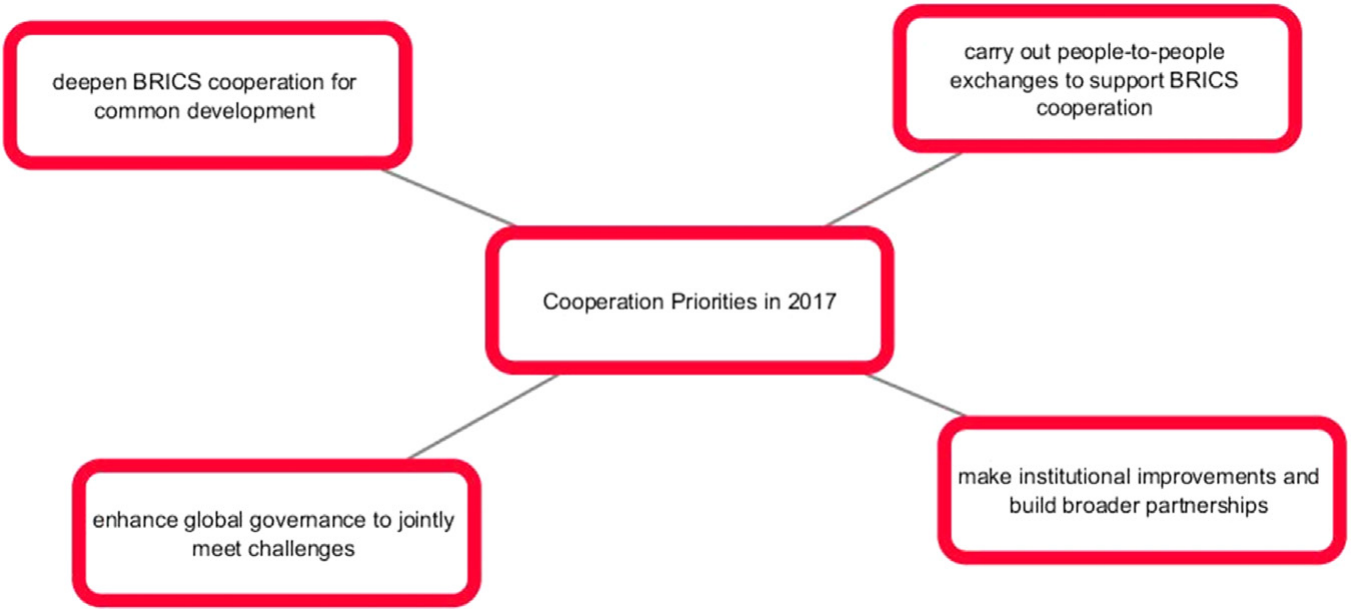
Cooperation priorities of Xiamen summit in 2017 [3].

We developed a special questionnaire to understand the media preferences of Russian people (namely, 274 participants) who follow the development of BRICS. Their professional fields (journalism, consulting, management, and marketing) resonate with BRICS agenda. Before their participation we requested their written consent to use their answers for our research (Table 1, Table 2, Table 3, Table 4).

**Table 1:** Media preferences.

**Table 2:** Preferred time and use.

**Table 3:** Preferred time duration of media use.

**Table 4:** Reasons for media preferences.

## 2 Experimental design, materials, and methods

We used the first data set in order to see the messages of two BRICS summits held by two different countries, Russia and China respectively. For the second data set we organized a special procedure which is as follows. To check the reliability of the developed questionnaire we offered pre-test variant of the questionnaire two weeks prior to the main survey [6]. At this stage, we invited people who were not a part of the main control group. Two weeks later we offered the same questionnaire to the same group of people, outside the main control group. We used the Guttmann scale of reproducibility to determine the reliability of our questionnaire. The result of this stage was 0,92 which corresponds to the high reliability of the developed questionnaire.

The questionnaire was given to respondents in a face-to-face interview. We offered the participants to answer the questions in their familiar interiors.

The results of the whole study show that BRICS audience in Russia receives information mainly from TV. Thus, a simple single-symbol Chinese-style design is more effective, because it provides a clear message at one single shot. However, in case the audience prefers the Internet format, it is advisable to offer a series of slides, according to the Russian model which engages people in browsing and reflecting on the delivered information.
