# Supplementary material for: BRICS soft power promotion: Dataset for media preference and use pattern among the Russian audience who follow the development of BRICS
Source: Data Brief. 2017 Dec 20;16:939–46. doi: 10.1016/j.dib.2017.12.004 (PMC5847624; doi:10.1016/j.dib.2017.12.004)
Supplement: Supplementary file 1 — Supplementary material [file mmc1.docx]

Conflict of Interest Form

By this form I state that my paper “**BRICS SOFT POWER PROMOTION: DATASET FOR MEDIA PREFERENCE AND USE PATTERN AMONG THE RUSSIAN AUDIENCE WHO FOLLOW THE DEVELOPMENT OF BRICS**

” doesn’t contain any conflict of interest. All acknowledgements are specified.
